# Mapping the nicking efficiencies of nickase R.BbvCI for side-specific LNA-substituted substrates using rolling circle amplification

**DOI:** 10.1038/srep32560

**Published:** 2016-09-01

**Authors:** Hua Wei, Guojie Zhao, Tianyu Hu, Suming Tang, Jiquan Jiang, Bo Hu, Yifu Guan

**Affiliations:** 1 Department of Biochemistry and Molecular Biology, China Medical University, #77 Puhe Road, Shenyang, Liaoning, 110122, China; 2 Animal Science and Veterinary Medicine College, Shenyang Agricultural University, Shenyang, Liaoning, 110866, China

## Abstract

We used a novel asymmetric cleavage analysis method based on rolling circle amplification (RCA) to determine the effects of LNA modification of substrate on the two subunits of R.BbvCI cleavage. We designed two sets of cleavage circular substrates by using two different ligation strategies and analyzed the single strand cleavage efficiency affected by different modification positions both from the cleaved strands and the uncleaved strands. Results showed that the effects of LNA on cleavage rates of modified strands and unmodified strands were both site-dependent. The Nb.BbvCI and Nt.BbvCI were affected by LNA modification in different way. Most of the modification positions showed strong inhibition of both of these two nickases cleavage. However, the modification in T3 position of bottom strand hardly affected both of the two nickases activities. The results suggested an intimated interaction between the two subunits of R.BbvCI, and the T3 position in bottom strand might be a less tight position which was hard to be disturbed.

Restriction endonucleases (REs) are extremely useful tool enzymes for DNA manipulation in molecular biology. The commonly used REs are type IIP REs which recognize palindromic sequences of 4 ~ 8 bps long and cleave precisely both strands inside the recognition sequences. On the other hand, type IIA, type IIS and some type IIT REs recognize non-palindromic sequences and make asymmetric cut on both strands either inside or outside the recognition sequences. In contrast to homodimeric type IIP REs these REs always consist of two different subunits. The two subunits behave differently for the two strands. However, each subunit alone has no cleavage activity^1^,^2^.

The asymmetric cleavage feature of heterodimeric restriction endonucleases leads to the innovative proposal of constructing artificial enzymes which can cleave only one strand of DNA duplex by abolishing the catalytic activity of one subunit of heterodimeric endonuclease. Thus, they are referred to as nickases or nicking endonucleases. Indeed, the strand-specific nickases have been successfully engineered by genetic mutation, including Bpu10I^3^,^4^, and BbvCI^5^,^6^. Depending on which subunit is inactivated, these nickases can make a selective cut on either the top strand or the bottom strand. In recent years, nickases have seen their increasing applications as useful tool enzymes. Combining with strand-displacing DNA polymerases, they can improve the isothermal amplification of DNA for highly sensitive nucleic acids detection^7^,^8^. They can nick DNA duplex for nucleic acids labeling^9^. Nicked or gapped DNA can also be prepared for DNA repairing^10^. However, the commercial available nicking enzymes are numbered. Exploiting the catalytic mechanism and engineering new nickases become urgent and attract more and more interests.

The fact that nicking one strand of DNA duplex requires co-presence of two different subunits suggests that specific interactions must be involved in the strand-specific nicking processes. Such interactions include those between two subunits, between each subunit and each strand. However, lack of well-defined tertiary structure data of nickases as well as adequate techniques for analysis of a nick on DNA duplex makes it very difficult to interpret the asymmetric cleavage mechanisms of nickases. Here, we proposed two strategies to overcome these difficulties.

First, we used a novel approach to analyze the single strand cleavage process of DNA duplex. Different from double-stranded DNA (dsDNA) break assay, analyzing individual single strand break of dsDNA is very important and irreplaceable for kinetic detail of RE catalysis, especially for nickase analysis. Traditional methods for such assay are based on electrophoresis^11^. Usually, the analyzed single strand of DNA duplex is labeled with radioisotope or fluorophore. After cleavage by REs, denaturing electrophoresis is employed to discriminate different lengths of ssDNA. The band shift during cleavage reaction indicates the relevant information of catalytic mechanisms and kinetics. These methods, however, are laborious, time-consuming and low-throughput. To overcome these technical drawbacks, we have developed a method to analyze the single strand break of DNA duplexes. The principle of this assay was based on rolling circle amplification (RCA), and was described briefly in Fig. 1. It consisted of a circular oligonucleotide (served as RCA template) and a short linear oligonucleotide (served as RCA primer). The duplex formed by the primer and the circular template contained the recognition sequence of a nickase. The cut site was located on the circular template. During incubation, nickase made a nick on the circular template, resulting in an abolished RCA. The more circular templates were cleaved, the less the RCA products were generated. Thus, by monitoring the fluorescent signal of RCA products, the nicking efficiency of a particular strand by nickase can be determined unambiguously (Fig. 1).

Secondly, we employed nucleotide analogs to substitute the DNA recognition sequences at specific sites. Such substitutions disturbed the contacts between DNA duplex and nickase, and inevitably altered the original molecular interactions. Thus, mapping the nicking activities for each site-specific substitution of the recognition sequences can be helpful to understand the catalytic mechanism. Nucleotide analogs exhibit many differences in chemical characteristics although they share structural similarities with native nucleotides. These distinctive features make them very useful in biology and medicine. They have been explored in antisense medicine for disease treatment^12^,^13^, and used in nucleic acid analysis for enhancing the target capture and non-target discrimination^14^. Their application to REs has progressed the revealing of the recognition and catalytic mechanisms of many REs^15^,^16^. Locked nucleic acid (LNA) is nucleotide analog having a bicyclic furanose unit of 2′-*O*, 4′-*C*-methylene-β-D-ribofuranosyl ring. It has the bridge structure that ‘locks’ the ribose in the C3′-endo sugar pucker conformation which is dominant in A-form DNA and RNA. When forming duplex, LNA has shown the ability to improve binding affinity towards complementary DNA or RNA strands^17^. It has been reported that LNA-substituted probes can enhance the discriminative power against the mismatches^18^,^19^,^20^,^21^,^22^,^23^,^24^,^25^,^26^. However, effects of LNA substitutions on nickases have been seldom explored. Here, we introduced LNA to probe the catalysis of a pair of nickases derived from R.BbvCI.

Natural R.BbvCI is a type IIA RE consisting of two subunits: R_1_ (31 kD) and R_2_ (32 kD). It recognizes the asymmetric sequence 5′-CC↓TCAGC-3′/5′-GC↓TGAGG-3′ (↓is the nicking site). Mutation in active center of each subunit of R.BbvCI gives rise to nickase N.BbvCI. The bioengineered mutant Nb.BbvCI (R_1_^+^R_2_^−^) can only make a nick on the bottom strand (BS) 5′-GC ↓TGAGG-3′, and the mutant Nt.BbvCI (R_1_^−^R_2_^+^) can nick the top strand (TS) 5′-CC↓TCAGC-3′^5^. We substituted the recognition sequence on both strands with LNA once a time, and measured the nicking activities of nickases Nt.BbvCI and Nb.BbvCI, respectively. We then proposed a preliminary insight into the complicated molecular interactions between the DNA duplex and the two subunits of R.BbvCI.

## Results

### Design of the analytic strategy

Overall mapping of LNA modification on N.BbvCI cleavage requires 14 site-specific substitutions on two septamer sequences of both top strand and bottom strand. The effects of these 14 modification positions on Nb.BbvCI and Nt.BbvCI should be determined respectively. Therefore, a systematic designing is important for the following experiments.

We designed two groups of oligonucleotides. One group can form DNA duplex with the other group. The first group was BS oligonucleotides of 46 nt long and contained bottom strand sequence (GC↓TGAGG). The septamer recognition sequence was modified by LNA at each site for bottom strand modification analysis. The modification sites were called LNA-G1b, C2b, T3b, G4b, A5b, G6b, G7b, respectively. The second group was TS oligonucleotides of 17 nt long and contained top strand sequence (CC↓TCAGC). The septamer recognition sequence was also modified by LNA at each site for top strand modification analysis. The modification sites were called LNA-C1t, C2t, T3t, C4t, A5t, G6t, C7t, respectively.

**Figure 2:**
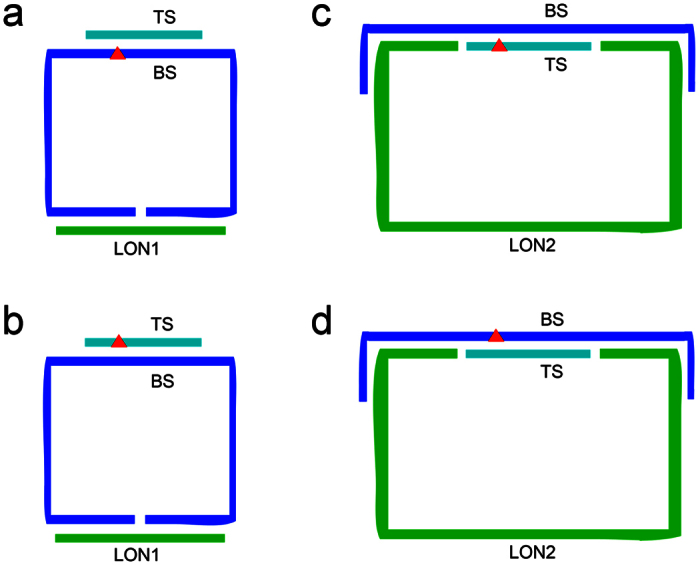
Preparation of circular templates for N.BbvCI nicking analysis. (**a**) LNA substitutions on bottom strand for Nb.BbvCI cleavage analysis. (**b**) LNA substitutions on top strand for Nb.BbvCI cleavage analysis. (**c**) LNA substitutions on top strand for Nt.BbvCI cleavage analysis. (**d**) LNA substitutions on bottom strand for Nt.BbvCI cleavage analysis. Red triangles represent LNA substitutions. LON1 and LON2 are oligonucleotides for ligation.

For Nb.BbvCI cleavage analysis, the BS oligonucleotides were circularized for RCA amplification by using another ligation splint oligonucleotide (termed as LON1). The circularized BS complemented with linear TS oligonucleotides to form DNA duplex substrates of Nb.BbvCI. The hybrid of unmodified BS and TS served as a standard reference of nicking efficiency under the native condition. Seven LNA-substituted BS pairing with the unmodified TS were used to analyze the intra-strand effects of LNA-substitutions (Fig. 2a), and seven LNA-substituted TS pairing with the unmodified BS were used to analyze the inter-strand effects of LNA-substitutions (Fig. 2b). Thus, we prepared 14 pairs of TS and BS for Nb.BbvCI nicking analysis. The detailed sequences were shown in Supplementary Table S1.

For Nt.BbvCI analysis, according to the principle, the TS oligonucleotides should be circularized. However, this was difficult in traditional splint-ligation way, since TS was shorter than BS oligonucleotides. In this case, we developed a novel splint (termed as LON2) which can hybridize with BS in a head-to-tail way and connect TS to form a circular template. The unmodified BS and TS DNA formed substrate, as a standard reference for nicking reaction. Seven LNA-substituted TS pairing with the unmodified BS were used to analyze the intra-strand effects of LNA-substitutions (Fig. 2c), and seven LNA-substituted BS pairing with the unmodified TS were used to analyze the inter-strand effects of LNA-substitutions (Fig. 2d). Consequently, 14 pairs of duplexes of TS and BS were prepared for Nt.BbvCI analysis. Since the nicking efficiencies were determined with respect to that of the unmodified BS-TS hybrid reference, the three-oligonucleotide circularization strategy would not create any inconsistence of the experimental results. The detailed sequences were shown in Supplementary Table S2.

### Nb.BbvCI cleavage of BS affected by LNA-substituted BS

**Figure 3:**
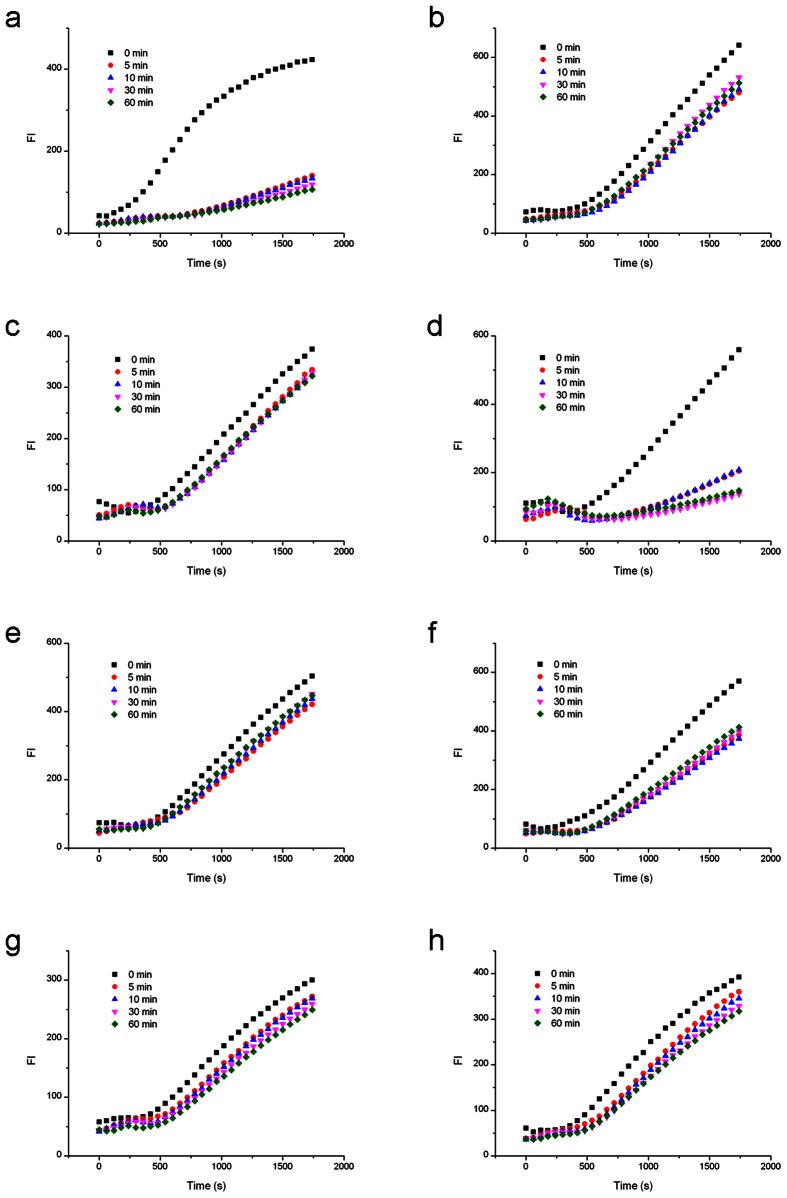
RCA analysis of Nb.BbvCI cleavage activities affected by LNA substitutions on the same strand to be nicked. (**a**) Cleavage of the unmodified bottom strand. (**b**) Cleavage of bottom strand by G1 modification. (**c**) Cleavage of bottom strand by C2 modification. (**d**) Cleavage of bottom strand by T3 modification. (**e**) Cleavage of bottom strand by G4 modification. (**f**) Cleavage of bottom strand by A5 modification. (**g**) Cleavage of bottom strand by G6 modification. (**h**) Cleavage of bottom strand by G7 modification.

First, we examined the Nb.BbvCI nicking on the bottom strands containing site-specific LNA substitutions (Fig. 2a). The bottom sequence of 5′-GC ↓TGAGG-3′ was substituted by LNA once a time. The uncleaved circular template of both unmodified control and seven modified substrates (0 min samples) presented effective RCA fluorescence signals, which confirmed the efficiency of circularization. During nicking process, RCA rates decreased to different extent for different modification positions (Fig. 3). As summerized in cleavage curve below, the cleavage of unmodified BS was very efficient. Cleavage efficiency increased abruptly once Nb.BbvCI was added, and the strand cleavage was finished in about 2 minutes. However, when the first nucleotide position (G1) was replaced by LNA-G1b, the cleavage was significantly inhibited. In the period of 60 minutes, the nicking efficiency was only around 20% in comparison with the unmodified DNA substrate. LNA modifications at other positions have also demonstrated similar strong inhibitory results. Interestingly, it seemed that LNA modification at LNA-T3b position (at the edge of the nick site) had very limited effect on the nicking activity, since the cleavage was almost as efficient as the unmodified recognition sequence. In contrast, cleavage at LNA-C2b position (another edge of the nick site) was strongly inhibited, indicating that the interactions of Nb.BbvCI with C2b and T3b positions were quite different. LNA substitutions at G4b, A5b, G6b and G7b considerably reduced the nicking efficiencies of Nb.BbvCI.

### Nb.BbvCI cleavage of BS affected by LNA-substituted TS

Inter-strand effects can be quite different from intra-strand effects. To test how the LNA substitutions on TS affected the complementary BS nicking by Nb.BbvCI, the LNA-substitutions were made on the top strand (Fig. 2b). Therefore the top sequence of the primer was modified by LNA, and then hybridized with the unmodified bottom sequence in the circular template.

**Figure 4:**
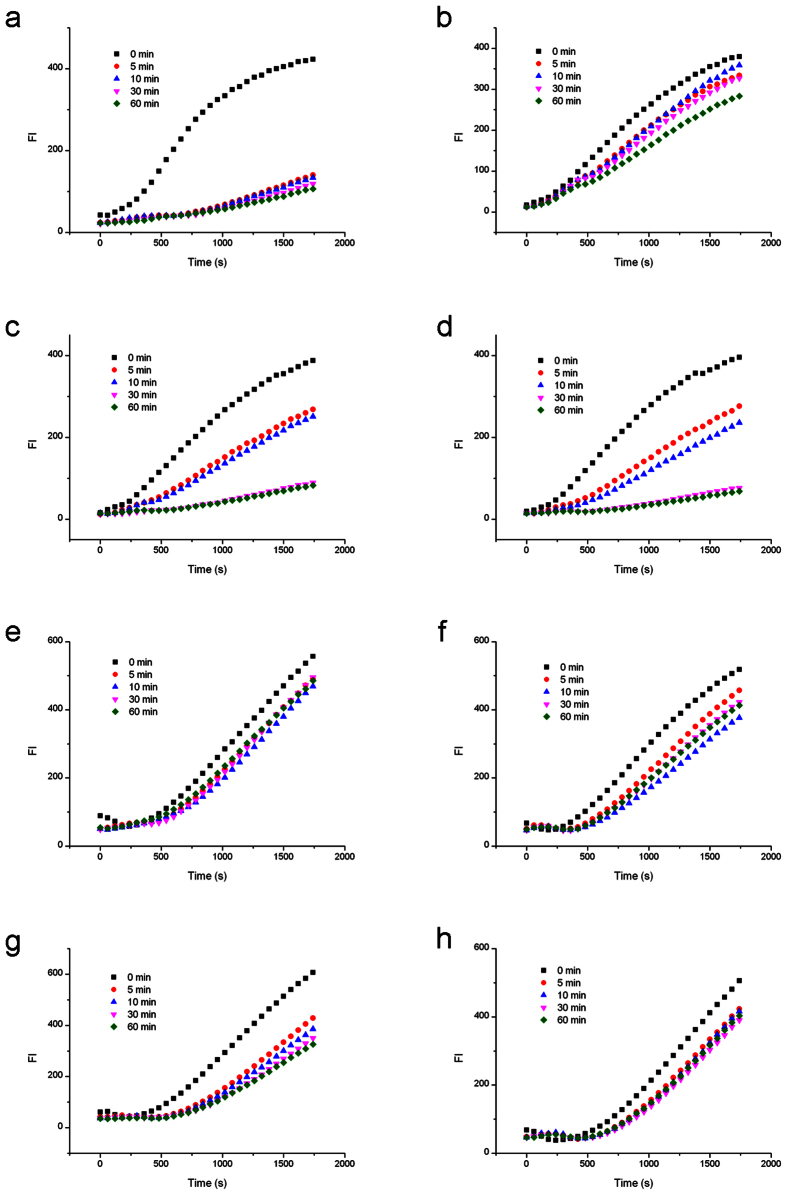
RCA analysis of Nb.BbvCI cleavage activities affected by the LNA substitutions on the complementary strand. (**a**) Cleavage affected by the unmodified top strand. (**b**) Cleavage affected by top strand with C1 modification. (**c**) Cleavage affected by top strand with C2 modification. (**d**) Cleavage affected by top strand with T3 modification. (**e**) Cleavage affected by top strand with C4 modification. (**f**) Cleavage affected by top strand with A5 modification. (**g**) Cleavage by top strand with G6 modification. (**h**) Cleavage affected by top strand with C7 modification.

In comparison with the native DNA substrate, the LNA-substitutions at seven positions of the top sequence 5′-CC↓TCAGC-3′ also showed different nicking efficiencies. As shown in cleavage curve (RCA fluorescence data see Fig. 4), LNA-C2t and LNA-T3t presented an obvious cleavage as efficient as that of unmodified DNA substrate after 60-minute cleavage. However, these cleavages took a longer time to finish, indicating that the enzyme and DNA substrate need a structural adaptation process to form an intermediate state for nicking. LNA-C4t and LNA-C7t showed complete inhibition of the nicking activity, and LNA-C1t, LNA-A5t and LNA-C6t exhibited more than 50% inhibitory effects. Interestingly, the effects of LNA-A5t and LNA-C6t, complementary to the bases around the nick, were different from that of LNA substitutions on the bottom sequence.

### Nt.BbvCI cleavage of TS affected by LNA-substituted TS

**Figure 5:**
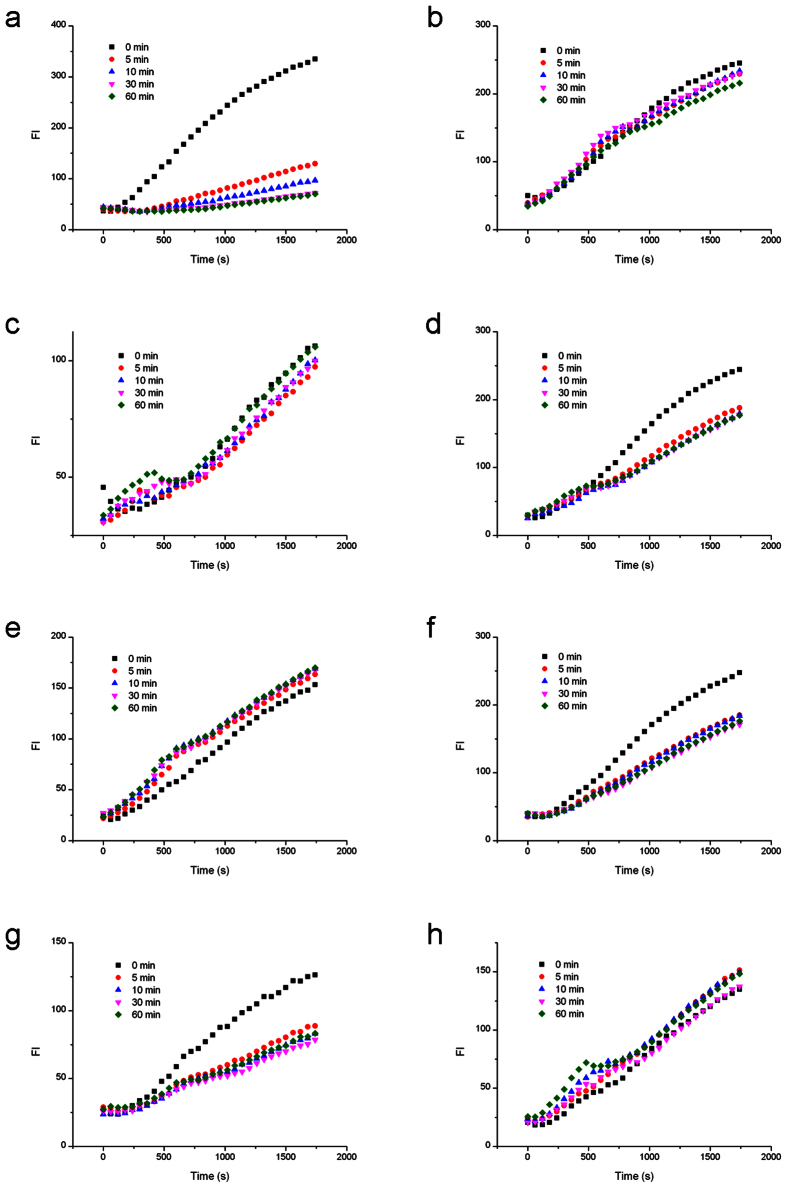
RCA analysis of Nt.BbvCI cleavage activities affected by the LNA substitutions on the same strand to be nicked. (**a**) Cleavage of the unmodified top strand. (**b**) Cleavage of top strand by C1 modification. (**c**) Cleavage of top strand by C2 modification. (**d**) Cleavage of top strand by T3 modification. (**e**) Cleavage of top strand by C4 modification. (**f**) Cleavage of top strand by A5 modification. (**g**) Cleavage of top strand by G6 modification. (**h**) Cleavage of top strand by C7 modification.

Different from Nb.BbvCI nicking the bottom strand, Nt.BbvCI only cleaves the top strand. Therefore, the TS was circularized for RCA amplification through a special splint LON2 (Fig. 2c,d). The uncleaved circular templates (0 min samples) all presented effective RCA signals, which verified the success of this three-oligonucleotide ligation strategy. After adding Nt.BbvCI, RCA rates were reduced during nicking process for unmodified DNA substrate (Fig. 5). In cases of LNA substitutions at T3t, A5t and G6t on the top strand, the nicking efficiencies of Nt.BbvCI were inhibited by about 50% after 60 minutes, whereas substitutions at C1t, C2t, C4t and C7t abolished almost completely the Nt.BbvCI activity. However, the effect of LNA-C2t was stronger than that of LNA-T3t substitution.

### Nt.BbvCI cleavage of TS affected by LNA-substituted BS

**Figure 6:**
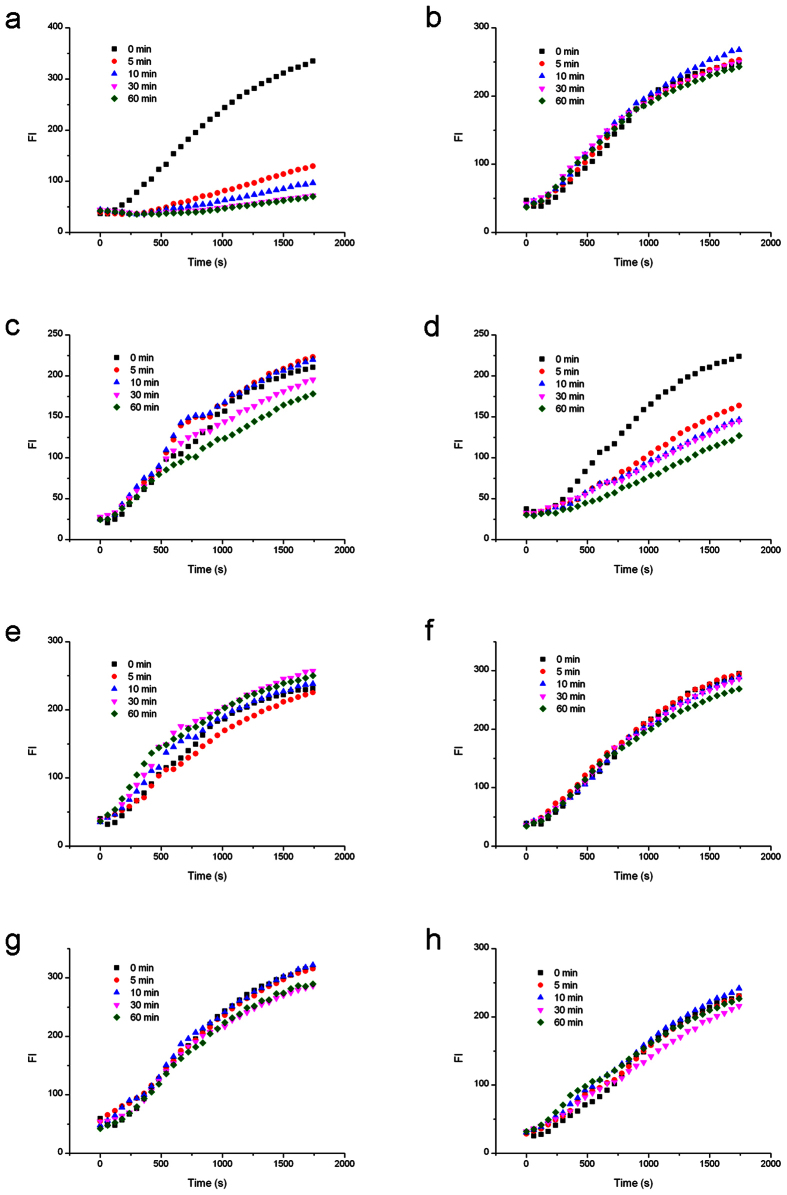
RCA analysis of Nt.BbvCI cleavage activities affected by the LNA substitutions on the complementary strand. (**a**) Cleavage affected by the unmodified bottom strand. (**b**) Cleavage affected by bottom strand with G1 modification. (**c**) Cleavage affected by bottom strand with C2 modification. (**d**) Cleavage affected by bottom strand with T3 modification. (**e**) Cleavage affected by bottom strand with G4 modification. (**f**) Cleavage affected by bottom strand with A5 modification. (**g**) Cleavage by bottom strand with G6 modification. (**h**) Cleavage affected by bottom strand with G7 modification.

**Cleavage curve** showed that LNA substitutions on the bottom strand influenced the cleavage of Nt.BbvCI on the top sequence (RCA fluorescence data see Fig. 6). LNA substitution at T3b position had a less effect on the nicking activity of Nt.BbvCI, reduced by ~30% in 60 minutes, whereas LNA-substitutions at other six positions strongly reduced the nicking efficiency.

## Discussion

RCA, developed from the DNA replication of circular viral genome, is an isothermal amplification process catalyzed by strand displacing DNA polymerases such as phi29 DNA polymerase. The mostly used RCA technique is for nucleic acids detection based on padlock probe strategy. The 5′- and 3′-termini of the padlock probe hybridize with the target of interest, and then the probe is circularized by DNA ligases. Using the circular padlock probe as RCA template and the target as RCA primer, phi29 DNA polymerase synthesizes ssDNA products with the tandem repeat of a sequence complementary to the padlock probe. RCA has become a versatile method for bioanalyses *in vitro* and *in situ*, such as SNP identification^27^,^28^, miRNA analysis^29^,^30^, mitochondrial DNA visualization in cells^31^, and protein detection^32^. Analysis of the increasing literatures of RCA showed that current RCA application is mainly restrained to the detection of nucleic acids. To expand its capabilities as a useful tool in biology and medicine, we utilized the RCA assay to explore the protein-DNA interactions. Utilizing the binding affinity of an allosteric repressor to its target DNA duplex, we succeeded in analyzing the L-Tryptophan in solution with high sensitivity and specificity^33^. We also measured the uneven cleavage of DNA duplexes by restriction endonucleases^34^. Here, we applied this fast, convenient, label-free and sensitive assay to mapping the nicking activities of N.BbvCI affected by nucleotide analog. Nickases have been employed in RCA to cleave amplified products with the purpose of enhancing RCA detection sensitivity^7^. However, analyzing nicking kinetics in detail by using RCA has rarely been reported. To verify this RCA-based method, we also conducted traditional electrophoresis analysis of all the 28 modifications. The electrophoresis results were accord well with the RCA results (See Supplementary Figs S1~S5). By comparing electrophoresis method with RCA assay in methodology, the former took 2 hours and three steps (gel preparation, electrophoresis, gel staining), while the RCA assay took only one step, and can be completed within 20 min. Moreover, RCA assay used 96-well plate, which was superior in high-throughput screening than gel electrophoresis.

Though challenged by increasing application demands, nickases converted from type IIA restriction endonucleases have been much less explored in comparison with type IIP restriction endonucleases. It is mainly due to the limited natural resources of nickases as well as lack of structural and catalytic information. Nucleotide analog substitution is an alternative strategy to supplement these shortcomings. Nucleotide analogs are nucleotides with modifications on bases, sugar ring or phosphate group. Depending upon the functional groups, nucleotide analogs demonstrate unusual properties different from their native counterparts in hydrophobicity, chemical reactivity, hydrogen-bond forming capability, electrostatic interaction, steric repulsion, etc. DNA duplexes with analog substitutions can change, directly or indirectly, the interactions with their partners (including enzymes, regulatory factors or other binding motifs). Nucleotide analog substitutions have been proved a powerful approach to uncover some aspects of kinetics and mechanisms of enzyme-catalyzed strand cleavage, gene expression regulation, DNA repairing and bioengineering manipulation.

**Figure 7:**
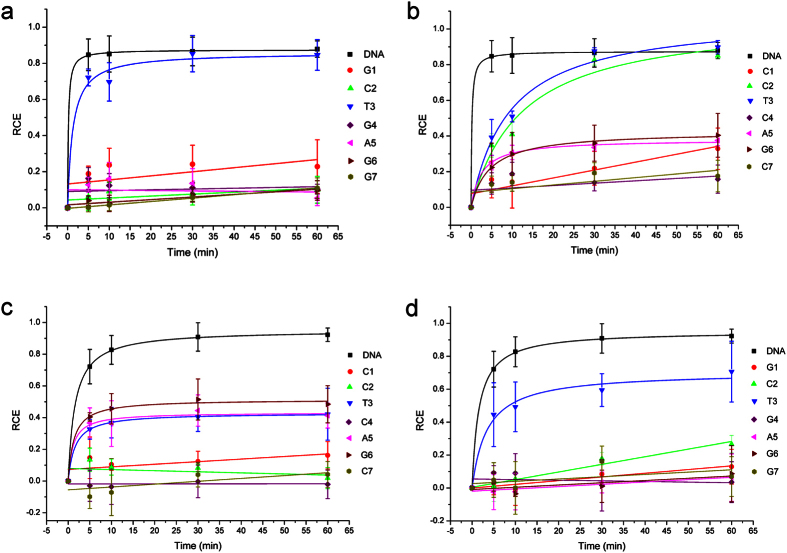
Cleavage efficiency of Nb.BbvCI or Nt.BbvCI affected by BS or TS modifications. (**a**) Nb.BbvCI cleavage activities affected by LNA substitutions on the same strand to be nicked (BS). (**b**) Nb.BbvCI cleavage activities affected by the LNA substitutions on the complementary strand (TS). (**c**) Nt.BbvCI cleavage activities affected by the LNA substitutions on the same strand to be nicked (TS). (**d**) Nt.BbvCI cleavage activities affected by the LNA substitutions on the complementary strand. DNA represents the unmodified strand. G1, C2, T3, G4, A5, G6 and G7 represent the LNA modified bottom strands. C1, C2, T3, C4, A5, G6 and C7 represent the LNA modified top strands. RCE represents the relative cleavage efficiency.

**Figure 8:**
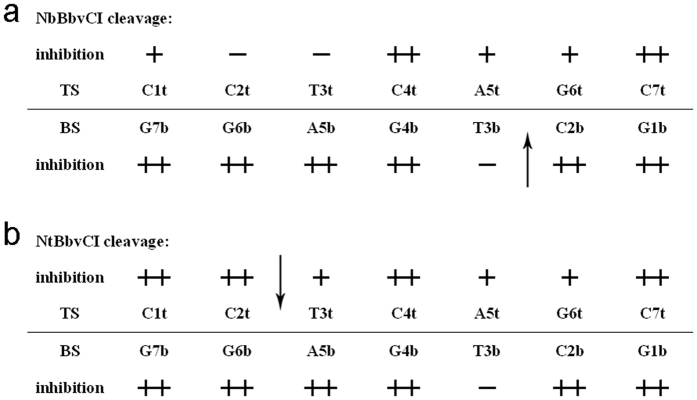
Position-dependent cleavage activities of Nb.BbvCI and Nt.BbvCI affected by LNA substitutions. Activity classification: ++: <30%; +: 30% ~ 70%; −: >70%. Arrows indicate the nicking sites.

From the cleavage curve of Fig. 7, we can derive Fig. 8, showing that the cleavage of N.BbvCI has been influenced remarkably by the site-specific LNA substitutions: significantly inhibited cleavage (>70%), moderately inhibited cleavage (between 30% and 70%), and little or no-affected (<30%). Comparison of two curves of native DNA substrate cleavage in Fig. 7a,c (labeled with ◾) suggested that nicking DNA duplexes by Nt.BbvCI and Nb.BbvCI was different. This observation was consistent with the study by Bellamy^6^. They have prepared the wide type R.BbvCI and two mutants of Nt.BbvCI and Nb.BbvCI, and obtained the turnover rates Kcat and rate constants of cleaving each strand. The rate constants of WT R.BbvCI to cleave the bottom and top sequences were 0.50 s^−1^ and 0.11 s^−1^, whereas these rate constants of Nb.BbvCI and Nt.BbvCI were 0.096 s^−1^ and 0.17 s^−1^, respectively.

The difference in rate constants must be due to the amino acid mutations in the putative catalytic center of each subunit. Sequence homology analyses demonstrated that the native R_1_ and R_2_ subunits of R.BbvCI had the KD-Xn-EVK and KD-Xn-ECK motifs in the putative catalytic centers, respectively (X being any amino acid residue), which was consistent with the conserved sequences KD-Xn-EXK of nickases Bpu10I, B1pI and Bsu36I^5^. To bioengineer Nt.BbvCI and Nb.BbvCI, R_1_ or R_2_ subunit was inactivated by mutating a key amino acid in its catalytic center. Nt.BbvCI had the mutated R_1_ subunit of …GVK … sequence, and Nb.BbvCI had the mutated R_2_ subunit of …GCK… sequence, respectively. The E → G mutations abolished the catalytic function of each subunit. It was apparent that EXK → GXK mutations from an acidic amino acid to a hydrophobic amino acid broke inevitably possible hydrogen bonds and coordinate bonds originally presented in the native R.BbvCI, and consequently altered the local structure around the catalytic center even the overall architecture of R.BbvCI might remain unchanged. The canonical B-form structure of DNA duplexes suggested that the two cleavage sites are only 3 bps apart and on the same side of the helical duplex. Therefore, the catalytic centers of R_1_ and R_2_ subunits are very close in space. In the case of Nt.BbvCI (R_1_^−^R_2_^+^), the GVK(R_1_) + ECK(R_2_) in the catalytic pocket behaved more hydrophobic than WT R.BbvCI (R_1_^+^R_2_^+^), and had no hydrogen-bond formation capability. On the other hand, the EVK(R_1_) + GCK(R_2_) motifs in the catalytic pocket of Nb.BbvCI (R_1_^+^R_2_^−^) lost the hydrogen-bond capability but was still hydrophilic and able to form the coordinate bond with Mg^2+^ ions as the WT R.BbvCI (R_1_^+^R_2_^+^)^2^.

Analyzing these cleavage curves provided hints of molecular interactions involved in the R.BbvCI cleavage. Figure 7a showed that the cleavage efficiencies of Nb.BbvCI were significantly repressed (more than 70%) by the LNA substitutions on BS except LNA-T3b. Though T3b was on the edge of the target nick site of Nb.BbvCI, LNA modification had a little effect on the nicking activity, suggesting that T3b was probably much less involved in phosphoester hydrolysis. Thus, LNA-T3b did not change the binding affinity of DNA duplex with Nb.BbvCI as well as the phosphoester bond hydrolysis. As known, there are six torsion angles (α, β, γ, δ, ε and ζ) along the nucleic acid backbone (-P-O5′-C5′-C4′-C3′-O3′-P-), and LNA substitution at T3b position exerted a much less influence on the distant -O3′-P- bond which was to be cleaved by nickase. Thus, it could be rationalized that T3b was probably used only for the sequence recognition.

In contrast, C2b on the other edge of the nick site behaved quite different from T3b: LNA-C2b inhibited cleavage of Nb.BbvCI completely (Fig. 7a). LNA substitution exerted a large impact directly on the -O3′-P- bond. Consequently, hydrogen-bond network and the intermediate state formation during the cleavage could be disturbed. It has been reported that LNA substitutions might cause the local structure changes of DNA duplexes^35^. LNA substitutions for G1b, G4b, A5b, G6b and G7b completely inhibited the Nb.BbvCI cleavage (Fig. 7a). These nucleotides were not around the nicking site, the inhibited hydrolyses were probably due to the reduced substrate binding affinity of the LNA-substituted duplex to the R_1_R_2_ heterodimer. These results revealed that nucleotides distant from the nick site also played an important role in determining the hydrolysis behavior.

Interestingly, LNA substitutions on the top sequence can also affect the Nb.BbvCI nicking process considerably. Cleavages of LNA-C2t and LNA-T3t by Nb.BbvCI were complete but slow (Fig. 7b). These results implied that the phosphoester bond may be fully hydrolyzed but the substrate binding affinity might be weak. LNA-C1t, LNA-A5t and LNA-G6t inhibited the Nb.BbvCI cleavage partially, while LNA-C4t and LNA-C7t did a complete inhibition (>70%). As described above, these repressed nicking activities could result from an unfavorable fit of the substrate into the binding domain of the R_1_R_2_ heterodimer, and breakage of the weak interactions between two subunits.

Based on the analyses above, it was not surprising to see that LNA substitutions at the top sequence influenced Nt.BbvCI cleavage. LNA-T3t, LNA-A5t and LNA-G6t moderately reduced the catalytic activity, and LNA-C1t, LNA-C2t, LNA-C4t and LNA-C7t inhibited the Nt.BbvCI cleavage significantly (Fig. 7c). Here, the inhibitory effect of LNA-C2t on the cleavage was stronger than that of LNA-T3t again, which was consistent with the observation that LNA substitution influenced the O3′-P bond more than the P-O5′ bond.

The intra-strand interactions were also observed for the Nt.BbvCI cleavage of the LNA substituted bottom sequence. Figure 7d showed that the shape of the LNA-T3b cleavage curve was similar to that of the DNA substrate but with a low cleavage efficiency, indicating that this retardation was probably caused by the reduced catalytic activity of Nt.BbvCI instead of the binding affinity. In contrast, LNA substitutions at other positions almost abolished the Nt.BbvCI activity.

In summary, we used a fast, convenient and high-throughput approach based on RCA principle to explore the interactions between DNA duplexes and proteins. As a practical application, we utilized it to investigate the single strand cleavage by nickases Nt.BbvCI and Nb.BbvCI. Furthermore, to identify the role of each individual nucleotide in the nicking events, we used nucleotide analog LNA to substitute specific nucleotides of the recognition sequences of R.BbvCI and examined the cleavage activities of each nickase. Experimental results showed that LNA substitutions on the top sequence can affect the nickase activity of Nb.BbvCI as well as that on the bottom sequence in a position-dependent fashion. Similar results have also been observed for Nt.BbvCI cleavage. Interestingly, two nucleotides on each edge of the nick affected the cleavage differently. LNA substitutions distant from the nick sites inhibited the nickase activity significantly, which could result from the weak interactions between R_1_ and R_2_ subunits. In addition, T3b modification had little effects on both Nb.BbvCI and Nt.BbvCI cleavage, which suggested a less tight position in enzyme-DNA interactions. These results and conclusions proved the potential of the RCA-based approach and nucleotide analog substitutions as versatile and useful practice to uncover molecular interactions of DNA duplex and proteins.

## Methods

### Oligonucleotides

DNA oligonucleotides at HPLC grade were purchased from Sangon Biotech Co. Ltd. (Shanghai, China). Their concentrations were determined by the absorption coefficiency of each sample. Nb.BbvCI, Nt.BbvCI and Phi29 DNA polymerase were purchased from NEB (Ipswith, MA). SYBR Green II was purchased from Invitrogen (Waltham, MA). T4 DNA Ligase was purchased from TaKaRa Biotechnology Co. Ltd. (Dalian, China). Site specific LNA-substituted oligonucleotides have also been prepared. LNA substitutions were made at seven positions of recognition sequences on the bottom strand or the top strand of R.BbvCI, respectively.

### Circular probe preparation

For Nb.BbvCI analysis, a free phosphate group was attached to the 5′-end of the bottom strand (BS). The 5′- and 3′-portions of BS (1 μM) were hybridized with 2 μM of ligation oligonucleotide 1 (LON1) in a head-to-tail fashion, and were covalently linked by T4 DNA ligase (175 U) at 16 °C for 60 min (Fig. 2a,b). Ligation buffer contains 50 mM Tris, 10 mM MgCl_2_, 5 mM DTT, and 0.1 mM ATP in 10 μl reaction systems (pH 8.0). For Nt.BbvCI analysis, the 5′-end of the top strand (TS) and ligation oligonucleotide 2 (LON2) were phosphorylated. The 5′- and 3′-portions of the LON2 (1 μM) and TS were hybridized with 2 μM of BS, and were covalently linked by T4 DNA ligase (175 U) at 16 °C for 60 min (Fig. 2c,d).

### Nickase cleavage

Circular template BS of 0.5 pmol was hybridized with 2 pmol top strand (TS) to form a short DNA duplex substrate of Nb.BbvCI. The cleavage reaction was carried out in 10 μl reaction buffer (pH 7.9) containing 50 mM potassium acetate, 20 mM Tris-acetate, 10 mM magnesium acetate, 100 μg/ml BSA. The cleavage was initiated by adding 5 U Nb.BbvCI. After incubation at 37 °C for 5, 10, 30 and 60 minutes, the cleavage reaction was stopped by heating at 90 °C for 20 min. For Nt.BbvCI analysis, circular template TS of 0.5 pmol was cleaved in 10 μl reaction buffer (pH 7.9) containing 50 mM potassium acetate, 20 mM Tris-acetate, 10 mM magnesium acetate, 100 μg/ml BSA. The cleavage was initiated by adding 5 U Nt.BbvCI. After incubation at 37 °C for 5, 10, 30 and 60 minutes, the cleavage was stopped by heating at 90 °C for 20 min.

### Rolling circle amplification

After cleavage, 0.25 pmol cleaved circular templates for Nb.BbvCI or Nt.BbvCI were added into RCA solution, respectively, which was composed of 50 mM Tris, 10 mM MgCl_2_, 10 mM (NH_4_)_2_SO_4_, 4 mM DTT (pH 7.5), SYBR Green II (1:10000), and phi29 DNA polymerase 3 U in 100 μl solution. Fluorescence signals were recorded on Microplate Reader (Infinite M200, Tecan, USA) with excitation wavelength at 480 nm and emission wavelength at 524 nm. The fluorescence emission was monitored for over ~20 min at 37 °C.

## Additional Information

**How to cite this article**: Wei, H. *et al*. Mapping the nicking efficiencies of nickase R.BbvCI for side-specific LNA-substituted substrates using rolling circle amplification. *Sci. Rep.* **6**, 32560; doi: 10.1038/srep32560 (2016).

## Supplementary Material

Supplementary Information

## Figures and Tables

**Figure 1:**
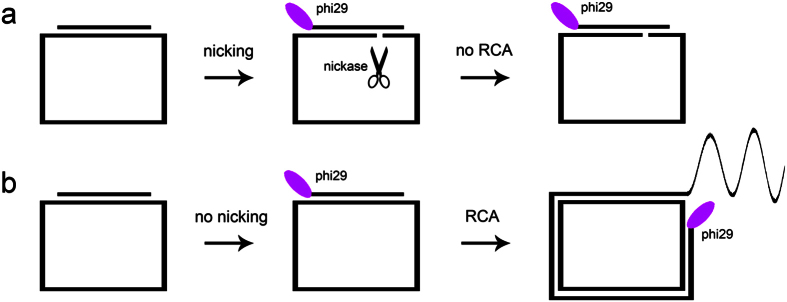
RCA-based assay for analyzing single strand cleavage by nickase. (**a**) Nicked circular template results in no RCA. (**b**) No nicked circular template results in RCA.
