# Supplementary material for: Mapping the nicking efficiencies of nickase R.BbvCI for side-specific LNA-substituted substrates using rolling circle amplification
Source: Sci Rep. 2016 Sep 1;6:32560. doi: 10.1038/srep32560 (PMC5007493; doi:10.1038/srep32560)
Supplement: Supplementary Information [file srep32560-s1.doc]

**Mapping the nicking efficiencies of nickase R.BbvCI for side-specific LNA-substituted substrates using rolling circle amplification**

Hua Wei^1,2,†^, Guojie Zhao^1,†^, Tianyu Hu^1^, Suming Tang^1^, Jiquan Jiang^1^, Bo Hu^1^, and Yifu Guan^1,*^

^1^Department of Biochemistry and Molecular Biology, China Medical University, #77 Puhe Road, Shenyang, Liaoning, 110122, China

^2^Animal Science and Veterinary Medicine College, Shenyang Agricultural University, Shenyang, Liaoning, 110866, China

^*^Correspondance and requests for materials should be addressed to Y.G. ([yfguan@mail.cmu.edu.cn](mailto:yfguan@mail.cmu.edu.cn))

^†^These authors contributed equally to this work

**Supplementary information**

**Supplementary Results**

**Denaturing PAGE analysis of LNA modifications on nicking.** We used DNA duplex formed by a long oligonucleotide (termed as L-BS/L-TS) and a short oligonucleotide (termed as S-TS/S-BS) as the substrates of Nb./Nt.BbvCI. Since nick hardly changes duplex mobility in native electrophoresis, denaturing electrophoresis is requisite to separate cleaved substrates in single-stranded form. L-BS and S-TS formed substrate for Nb.BbvCI. After nicking, 46 nt long L-BS broke into 30 nt and 16 nt long oligonucleotides (Figure S1a). The former was clearly shown in Figure S1b (Lane 2~3), and the latter was hard to be discriminated from S-TS which was 17 nt long. For Nt.BbvCI cleavage, L-TS and S-BS were used as substrate. Similar results were found in Figure S1b (Lane 4~5). Therefore, we chose 46 nt band as nicking substrate and 30 nt band as nicking product to monitor the nicking process. In addition, we also found that S-TS was more slightly silver-stained than S-BS did, which might be related with their sequences (Figure S1b).

For Nb.BbvCI cleavage of L-BS affected by BS modification, compared with unmodified DNA control, only T3b was cleaved obviously. Other modifications all strongly inhibited cleavage (Figure S2). This was accord with RCA results very well. For Nb.BbvCI cleavage of L-BS affected by TS modification, C2t and T3t presented little inhibition. C1t, A5t and G6t inhibited nicking to some extent. C4t and C7t almost inhibited cleavage completely (Figure S3). This also verified the results of RCA analysis.

For Nt.BbvCI cleavage of L-TS affected by TS modification, T3t, A5t and G6t only inhibited cleavage partly, while C1t, C2t, C4t and C7t inhibited cleavage almost completely (Figure S4), which was the same with RCA results. For Nt.BbvCI cleavage of L-TS affected by BS modification, T3b presented fast cleavage, while other modifications behaved strongly inhibition (Fig S5), which was also accord with RCA results. Summarizing all these PAGE results, we can derive the same table in Figure 8. Therefore, PAGE results verified the RCA-based method.

**Supplementary data**

**Figures:**


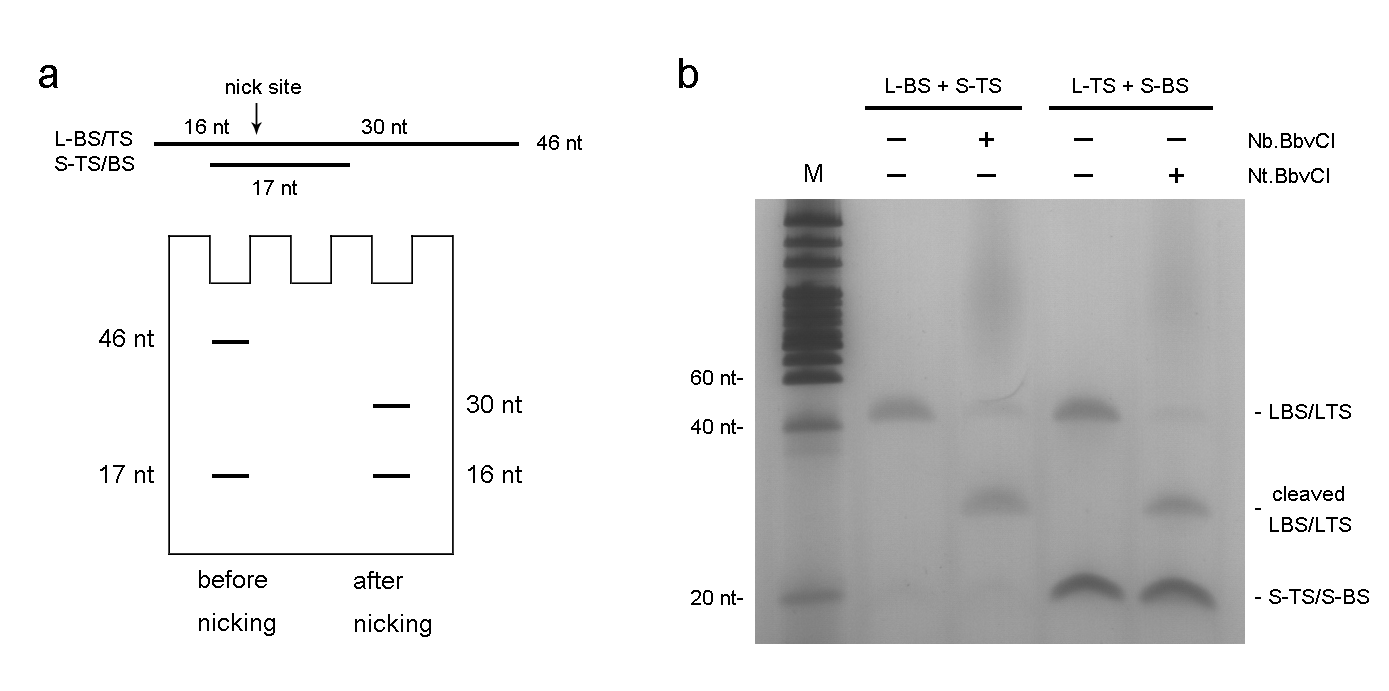


**Figure S1. Denaturing PAGE analysis of duplex nicking.** (a) Schematic of the DNA substrates and the nicked products for PAGE analysis. (b) Lanes 2 and 3: DNA duplex formed by L-BS and S-TS, nicked by Nb.BbvCI; Lanes 4 and 5: DNA duplex formed by L-TS and S-BS, nicked by Nt.BbvCI.


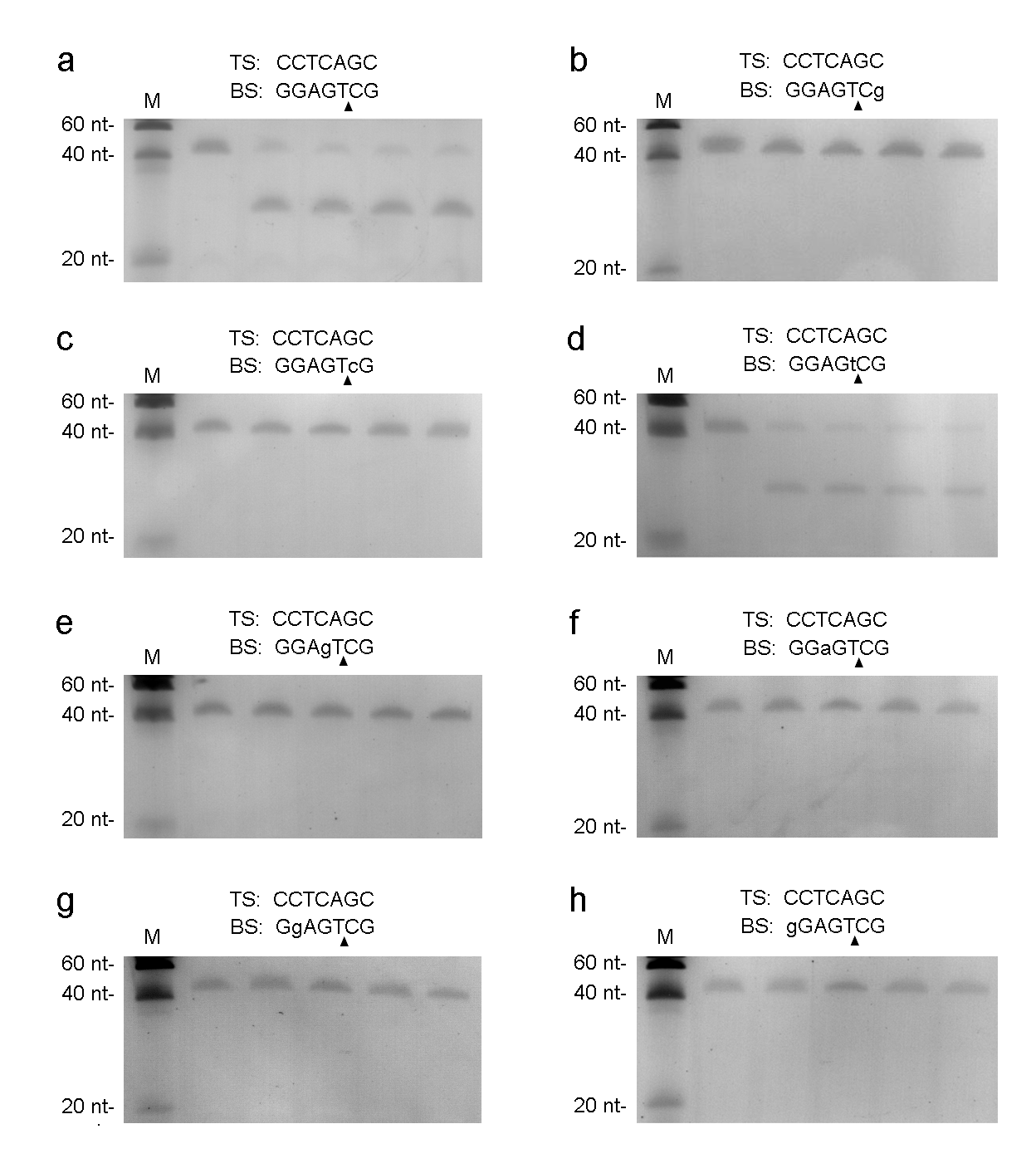


**Figure S2.** **Denaturing PAGE analysis of Nb.BbvCI cleavage activities affected by LNA substitutions on the same strand to be nicked (BS).** (a) Cleavage of the unmodified bottom strand. (b) Cleavage of bottom strand by G1 modification. (c) Cleavage of bottom strand by C2 modification. (d) Cleavage of bottom strand by T3 modification. (e) Cleavage of bottom strand by G4 modification. (f) Cleavage of bottom strand by A5 modification. (g) Cleavage of bottom strand by G6 modification. (h) Cleavage of bottom strand by G7 modification. Duplex recognition sequences are given above the gels. Lower case letters represent LNA-modifications. Black triangles represent nick sites.


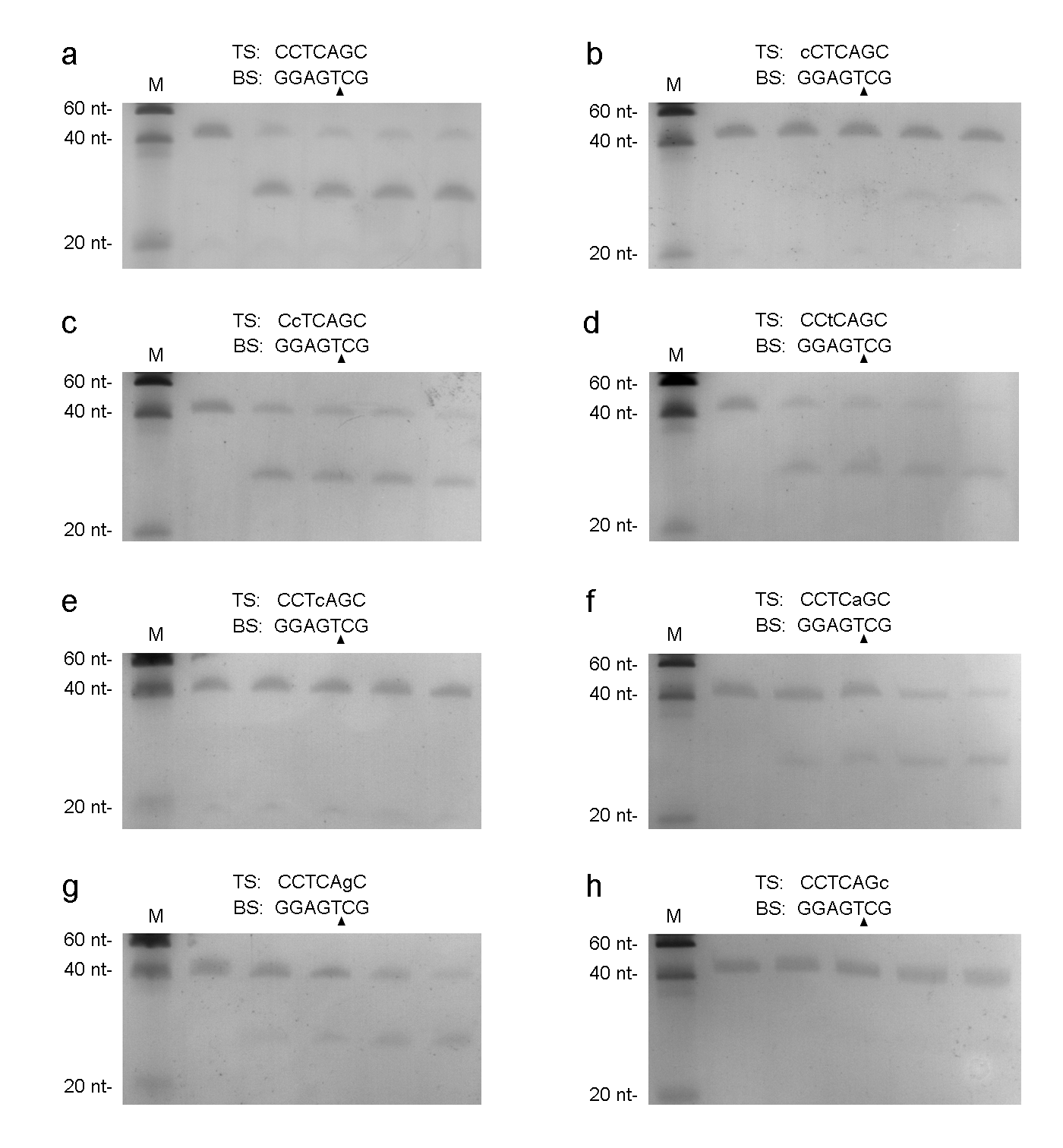


**Figure S3. Denaturing PAGE analysis of Nb.BbvCI cleavage activities affected by the LNA substitutions on the complementary strand (TS).** (a) Cleavage affected by the unmodified top strand. (b) Cleavage affected by top strand with C1 modification. (c) Cleavage affected by top strand with C2 modification. (d) Cleavage affected by top strand with T3 modification. (e) Cleavage affected by top strand with C4 modification. (f) Cleavage affected by top strand with A5 modification. (g) Cleavage by top strand with G6 modification. (h) Cleavage affected by top strand with C7 modification. Duplex recognition sequences are given above the gels. Lower case letters represent LNA-modifications. Black triangles represent nick sites.


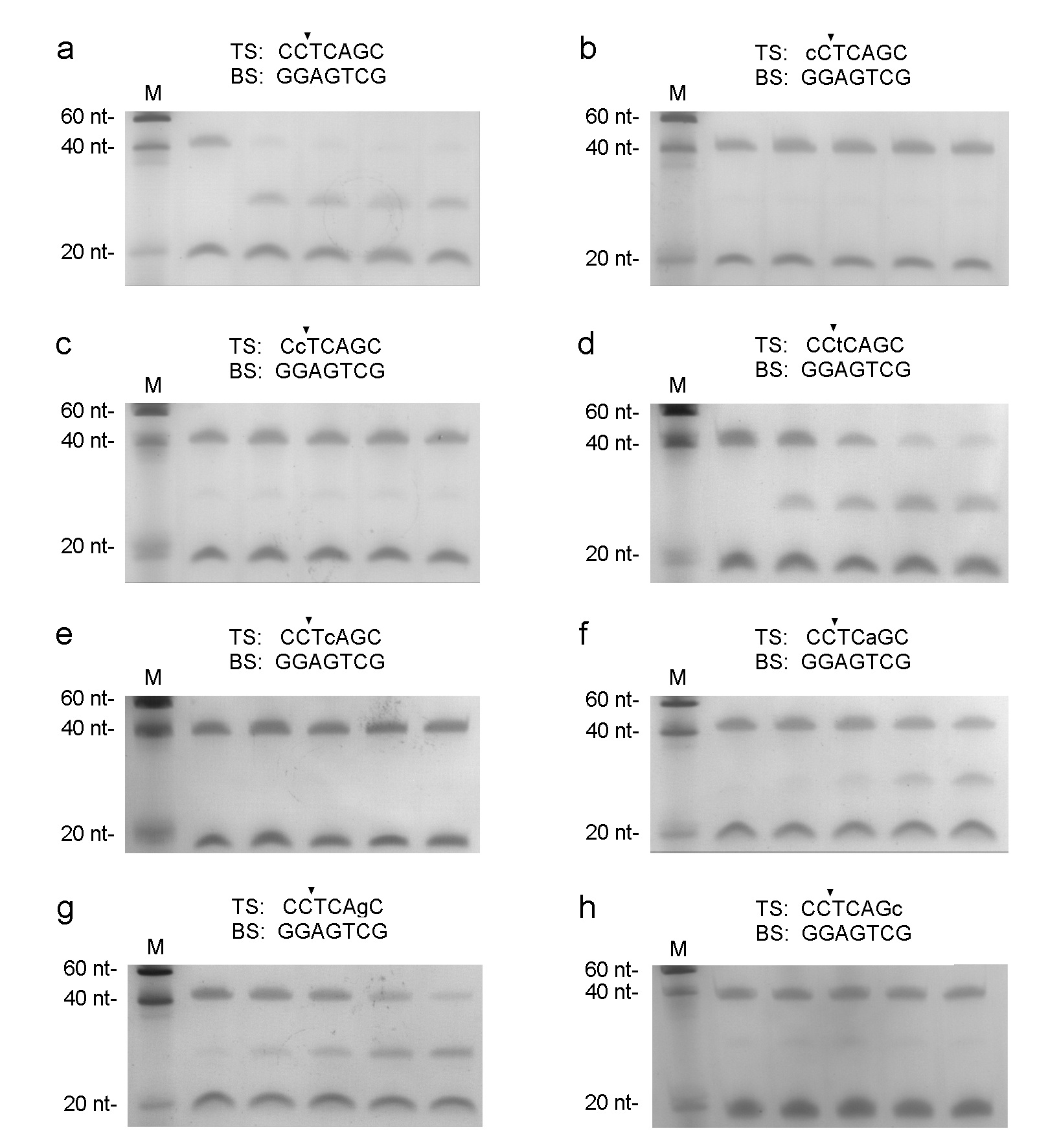


**Figure S4. Denaturing PAGE analysis of Nt.BbvCI cleavage activities affected by the LNA substitutions on the same strand to be nicked (TS).** (a) Cleavage of the unmodified top strand. (b) Cleavage of top strand by C1 modification. (c) Cleavage of top strand by C2 modification. (d) Cleavage of top strand by T3 modification. (e) Cleavage of top strand by C4 modification. (f) Cleavage of top strand by A5 modification. (g) Cleavage of top strand by G6 modification. (h) Cleavage of top strand by C7 modification. Duplex recognition sequences are given above the gels. Lower case letters represent LNA-modifications. Black triangles represent nick sites.


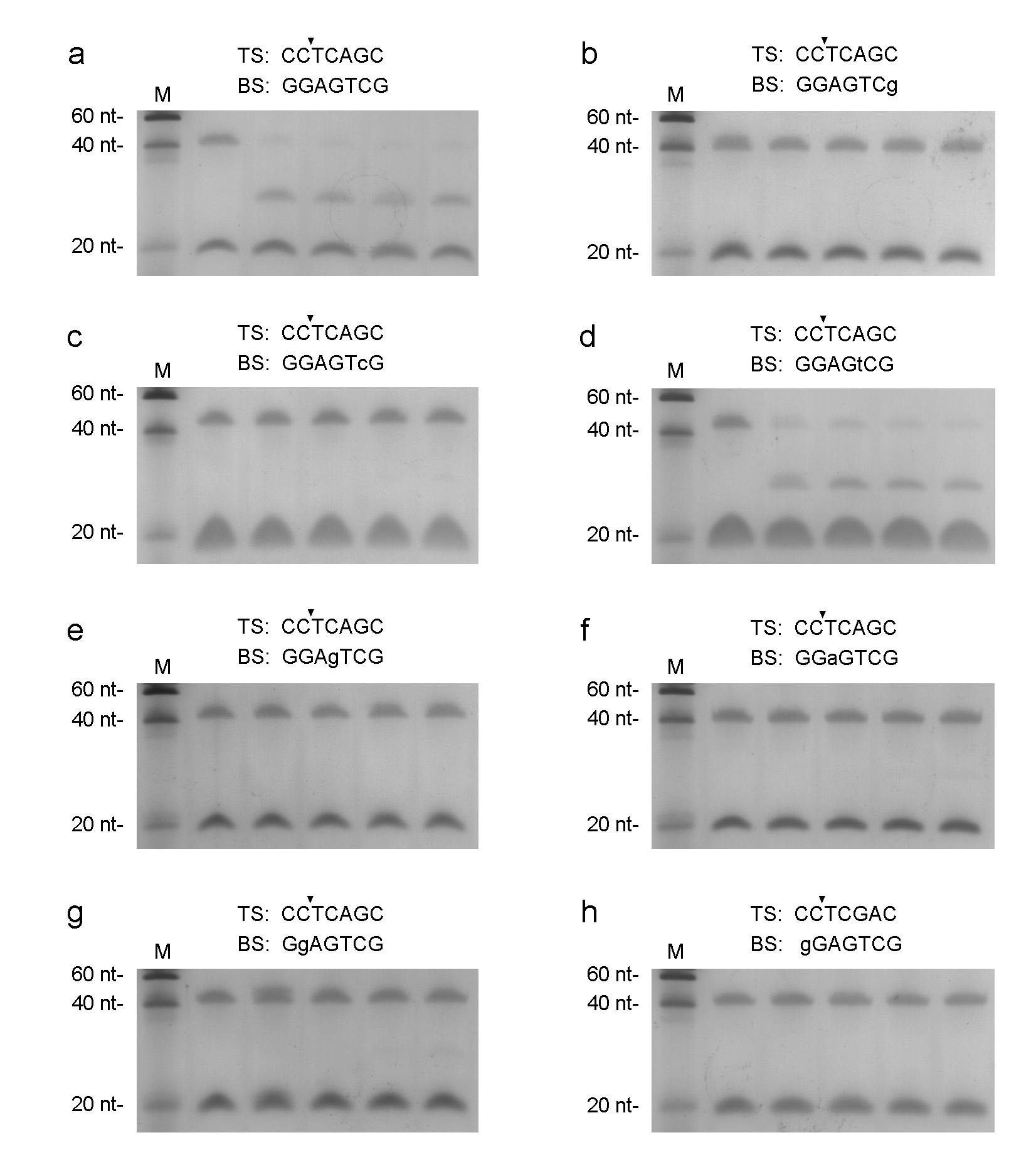


**Figure S5. Denaturing PAGE analysis of Nt.BbvCI cleavage activities affected by the LNA substitutions on the complementary strand (BS).** (a) Cleavage affected by the unmodified bottom strand. (b) Cleavage affected by bottom strand with G1 modification. (c) Cleavage affected by bottom strand with C2 modification. (d) Cleavage affected by bottom strand with T3 modification. (e) Cleavage affected by bottom strand with G4 modification. (f) Cleavage affected by bottom strand with A5 modification. (g) Cleavage by bottom strand with G6 modification. (h) Cleavage affected by bottom strand with G7 modification. Duplex recognition sequences are given above the gels. Lower case letters represent LNA-modifications. Black triangles represent nick sites.

**Tables**

**Table S1.** Oligonucleotides for Nb.BbvCI cleavage analyses by RCA.

| Name | Sequence |
| --- | --- |
| BS-DNA | 5’-p-ATACGCATACCTGTGCTGAGGTGGCTAAAAGCACACGCACGGAGAC |
| BS-G1 | 5’-p-ATACGCATACCTGTgCTGAGGTGGCTAAAAGCACACGCACGGAGAC |
| BS-C2 | 5’-p-ATACGCATACCTGTGcTGAGGTGGCTAAAAGCACACGCACGGAGAC |
| BS-T3 | 5’-p-ATACGCATACCTGTGCtGAGGTGGCTAAAAGCACACGCACGGAGAC |
| BS-G4 | 5’-p-ATACGCATACCTGTGCTgAGGTGGCTAAAAGCACACGCACGGAGAC |
| BS-A5 | 5’-p-ATACGCATACCTGTGCTGaGGTGGCTAAAAGCACACGCACGGAGAC |
| BS-G6 | 5’-p-ATACGCATACCTGTGCTGAgGTGGCTAAAAGCACACGCACGGAGAC |
| BS-G7 | 5’-p-ATACGCATACCTGTGCTGAGgTGGCTAAAAGCACACGCACGGAGAC |
| TS-DNA | 5’-p-TAGCCACCTCAGCACAG |
| TS-C1 | 5’-p-TAGCCAcCTCAGCACAG |
| TS-C2 | 5’-p-TAGCCACcTCAGCACAG |
| TS-T3 | 5’-p-TAGCCACCtCAGCACAG |
| TS-C4 | 5’-p-TAGCCACCTcAGCACAG |
| TS-A5 | 5’-p-TAGCCACCTCaGCACAG |
| TS-G6 | 5’-p-TAGCCACCTCAgCACAG |
| TS-C7 | 5’-p-TAGCCACCTCAGcACAG |
| LON1 | 5’-GTATGCGTATGTCTCCGTGC |

* The recognition sequences of R.BbvCI are underlined. LNA nucleotides are in the lower case.

**Table S2.** Oligonucleotides for Nt.BbvCI cleavage analyses by RCA.

| Name | Sequence |
| --- | --- |
| BS-G1 | 5’-p-ATACGCATACCTGTgCTGAGGTGGCTAAAAGCACACGCACGGAGAC |
| BS-C2 | 5’-p-ATACGCATACCTGTGcTGAGGTGGCTAAAAGCACACGCACGGAGAC |
| BS-T3 | 5’-p-ATACGCATACCTGTGCtGAGGTGGCTAAAAGCACACGCACGGAGAC |
| BS-G4 | 5’-p-ATACGCATACCTGTGCTgAGGTGGCTAAAAGCACACGCACGGAGAC |
| BS-A5 | 5’-p-ATACGCATACCTGTGCTGaGGTGGCTAAAAGCACACGCACGGAGAC |
| BS-G6 | 5’-p-ATACGCATACCTGTGCTGAgGTGGCTAAAAGCACACGCACGGAGAC |
| BS-G7 | 5’-p-ATACGCATACCTGTGCTGAGgTGGCTAAAAGCACACGCACGGAGAC |
| TS-C1 | 5’-p-TAGCCAcCTCAGCACAG |
| TS-C2 | 5’-p-TAGCCACcTCAGCACAG |
| TS-T3 | 5’-p-TAGCCACCtCAGCACAG |
| TS-C4 | 5’-p-TAGCCACCTcAGCACAG |
| TS-A5 | 5’-p-TAGCCACCTCaGCACAG |
| TS-G6 | 5’-p-TAGCCACCTCAgCACAG |
| TS-C7 | 5’-p-TAGCCACCTCAGcACAG |
| LON2 | 5’-p-GTATGCGTATATCCGGATATAGTTCCTCCTGTCTCCGTGCGTGTGCTTT |

*The recognition sequence of Nb./Nt.BbvCI is underlined. LNA nucleotides are in the lower case.

**Table S3.** Oligonucleotides for Nb.BbvCI cleavage analyses by denaturing PAGE.

| Name | Sequence |
| --- | --- |
| L-BS-DNA | 5’-ATACGCATACCTGTGCTGAGGTGGCTAAAAGCACACGCACGGAGAC |
| L-BS-G1 | 5’-ATACGCATACCTGTgCTGAGGTGGCTAAAAGCACACGCACGGAGAC |
| L-BS-C2 | 5’-ATACGCATACCTGTGcTGAGGTGGCTAAAAGCACACGCACGGAGAC |
| L-BS-T3 | 5’-ATACGCATACCTGTGCtGAGGTGGCTAAAAGCACACGCACGGAGAC |
| L-BS-G4 | 5’-ATACGCATACCTGTGCTgAGGTGGCTAAAAGCACACGCACGGAGAC |
| L-BS-A5 | 5’-ATACGCATACCTGTGCTGaGGTGGCTAAAAGCACACGCACGGAGAC |
| L-BS-G6 | 5’-ATACGCATACCTGTGCTGAgGTGGCTAAAAGCACACGCACGGAGAC |
| L-BS-G7 | 5’-ATACGCATACCTGTGCTGAGgTGGCTAAAAGCACACGCACGGAGAC |
| S-TS-DNA | 5’-TAGCCACCTCAGCACAG |
| S-TS-C1 | 5’-TAGCCAcCTCAGCACAG |
| S-TS-C2 | 5’-TAGCCACcTCAGCACAG |
| S-TS-T3 | 5’-TAGCCACCtCAGCACAG |
| S-TS-C4 | 5’-TAGCCACCTcAGCACAG |
| S-TS-A5 | 5’-TAGCCACCTCaGCACAG |
| S-TS-G6 | 5’-TAGCCACCTCAgCACAG |
| S-TS-C7 | 5’-TAGCCACCTCAGcACAG |

* The recognition sequences of R.BbvCI are underlined. LNA nucleotides are in the lower case.

**Table S4.** Oligonucleotides for Nt.BbvCI cleavage analyses by denaturing PAGE.

| Name | Sequence |
| --- | --- |
| L-TS-DNA | 5’-ATACGCATACCTGTCCTCAGCTGGCTAAAAGCACACGCACGGAGAC |
| L-TS-C1 | 5’-ATACGCATACCTGTcCTCAGCTGGCTAAAAGCACACGCACGGAGAC |
| L-TS-C2 | 5’-ATACGCATACCTGTCcTCAGCTGGCTAAAAGCACACGCACGGAGAC |
| L-TS-T3 | 5’-ATACGCATACCTGTCCtCAGCTGGCTAAAAGCACACGCACGGAGAC |
| L-TS-C4 | 5’-ATACGCATACCTGTCCTcAGCTGGCTAAAAGCACACGCACGGAGAC |
| L-TS-A5 | 5’-ATACGCATACCTGTCCTCaGCTGGCTAAAAGCACACGCACGGAGAC |
| L-TS-G6 | 5’-ATACGCATACCTGTCCTCAgCTGGCTAAAAGCACACGCACGGAGAC |
| L-TS-C7 | 5’-ATACGCATACCTGTCCTCAGcTGGCTAAAAGCACACGCACGGAGAC |
| S-BS-DNA | 5’-TAGCCAGCTGAGGACAG |
| S-BS-G1 | 5’-TAGCCAgCTGAGGACAG |
| S-BS-C2 | 5’-TAGCCAGcTGAGGACAG |
| S-BS-T3 | 5’-TAGCCAGCtGAGGACAG |
| S-BS-G4 | 5’-TAGCCAGCTgAGGACAG |
| S-BS-A5 | 5’-TAGCCAGCTGaGGACAG |
| S-BS-G6 | 5’-TAGCCAGCTGAgGACAG |
| S-BS-G7 | 5’-TAGCCAGCTGAGgACAG |

* The recognition sequences of R.BbvCI are underlined. LNA nucleotides are in the lower case.

**Supplementary Methods**

**Oligonucleotides.** Four groups of oligonucleotides were designed for denaturing PAGE analysis. Long ON (46 nt) bearing BS sequence (L-BS) and short ON (17 nt) bearing TS sequence (S-TS) form duplex for Nb.BbvCI analysis. Long ON (46 nt) bearing TS sequence (L-TS) and short ON (17 nt) bearing BS sequence (S-BS) form duplex for Nt.BbvCI analysis. The TS and BS sequences were modified by LNA site by site. HPLC grade oligonucleotides were purchased from Sangon Biotech Co. Ltd. (Shanghai, China). Their sequences were listed in Table S3 and Table S4.

**Nickase cleavage.** For Nb.BbvCI cleavage analysis, 0.5 pmol L-BS complemented with 1 pmol S-TS was cleaved in 10 μl reaction system containing 20 mM Tris-acetate, 50 mM potassium acetate, 10mM magnesium acetate, 100 μg/ml BSA, pH 7.9, 5 U Nb.BbvCI for 0, 5, 10, 30, 60 min, respectively. Modified L-BS with unmodified S-TS was for intra-strand analysis. Unmodified L-BS with modified S-TS was for inter-strand analysis. Duplex formed by unmodified L-BS and S-TS was DNA reference. For Nt.BbvCI cleavage analysis, L-TS and S-BS were used to form substrate.

**PAGE analysis.** 18% denaturing acrylamide gel containing 7 M urea was prepared. The cleavage reaction samples were loaded into the gel. Electrophoresis was carried out under constant 200 V for 1 hour. The gel was stained by silver staining method for about 30 min, as described previously[^1^](#_ENREF_1).

References

1. Zhao, G. & Guan, Y. Polymerization behavior of Klenow fragment and Taq DNA polymerase in short primer extension reactions. *Acta Biochim Biophys Sin (Shanghai)* **42**, 722-728 (2010).
